# Growth and development of two predator species fed a diet of genetically engineered mosquitoes

**DOI:** 10.1186/s13071-025-06987-6

**Published:** 2025-08-20

**Authors:** Claire M. Egan, Lisa Chamberland, Robert E. Ditter, Melina Campos, Fatima Batchelor, Aleena Bosky, Christine H. Coleman, Andrew J. Goffinet, Ariana Hosseini, Morgan Kammersgard, Brian Leetakubuulidde, Danspaid P. Mabuka, Ivan Mulongo Mugeni, Gregory C. Lanzaro

**Affiliations:** 1 Vector Genetics Laboratory, Department of Pathology, Microbiology and Immunology, UC Davis, Davis, CA USA; 2 Department of Biological Sciences, State University of New York at Buffalo, Buffalo, NY USA

**Keywords:** Genetically engineered mosquitoes, Gene drive, Malaria, Nontarget organisms, GMO environmental impact

## Abstract

**Background:**

Genetically engineered mosquitoes (GEMs) with gene drives have been developed for malaria control but remain untested in natural environments. Upon release, GEMs are expected to modify or replace wild-type counterparts, potentially uniquely interacting with nontarget organisms (NTOs). Concerns exist over possible negative effects on NTOs and broader ecological harm. Predators consuming GEMs represent a group that interacts closely with these modified mosquitoes.

**Methods:**

Here, we examine the effect of GEM and wild-type *Anopheles coluzzii* diets on the growth of two predator species: the aquatic mosquitofish (*Gambusia affinis*) and the terrestrial bold jumping spider (*Phidippus audax*). *Gambusia affinis* was fed lyophilized gravid mosquitoes, and growth was measured using length and mass. *Phidippus audax* was fed live semi-gravid mosquitoes, with growth tracked via eye size, body size, and mass.

**Results:**

No adverse effects were found in either predator species fed GEM diets. *Gambusia affinis* showed no significant growth differences between diet groups. However, *P. audax* that were fed GEMs consumed more mosquitoes, grew larger, and matured faster.

**Conclusions:**

Differences in predator growth rate suggest that GEMs’ nutritional content is similar to that of wild-type mosquitoes, but that they may be more vulnerable to predation. Further research is needed to explore whether GEM visual or behavioral traits increase their susceptibility to predators.

**Graphical abstract:**

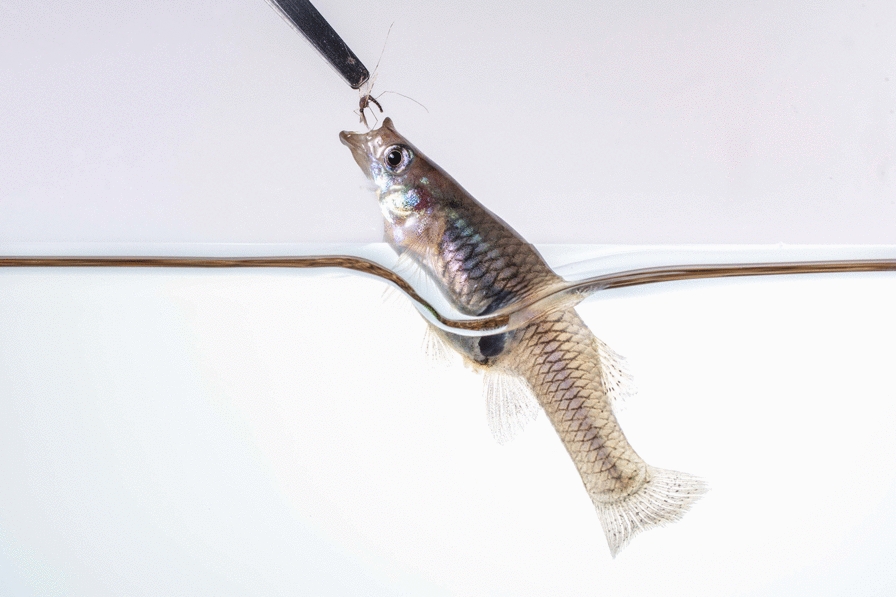

**Supplementary information:**

The online version contains supplementary material available at 10.1186/s13071-025-06987-6.

## Background

In 2023, there were an estimated 263 million malaria cases and 597,000 malaria-related deaths; 94% of cases and 95% of deaths occurred in the World Health Organization (WHO) African Region, where members of the *Anopheles gambiae* species complex are primary vectors [1]. A variety of conventional methods to combat malaria focus on vector control. These include indoor residual spraying, insecticide-treated bed nets, larval source management, and introduction of biological control agents [2–4]. Despite widespread application of these conventional methods, progress toward malaria elimination has slowed [5]. Genetically engineered mosquitoes (GEMs) have been proposed as a next-generation technological tool to be implemented alongside existing methods to improve malaria eradication efforts [6]. The University of California Malaria Initiative (UCMI) has engineered an *Anopheles coluzzii* mosquito with a genetic construct that couples *Plasmodium falciparum* single-chain antibodies with a gene-drive system. *An. coluzzii* is the UCMI target species because it is a major vector of malaria throughout its range in Western and Central Africa [7]. UCMI GEMs spread malaria resistance through natural *An. coluzzii* populations at a rate far higher than is expected with typical Mendelian inheritance [8].

GEMs, such as the one created by UCMI, are capable of spreading by mating with wild-type (WT) individuals to produce close to 100% malaria-resistant progeny, thus modifying an existing target population rather than eliminating it [9]. This approach, known as population modification, does not remove the mosquito from the environment or leave its ecological niche open. These characteristics contribute to reducing unintended environmental consequences associated with a population suppression approach, which removes the target mosquito from the environment [10]. The UCMI GEM is expected to persist for multiple generations at a field site postrelease; therefore, it will regularly interact with nontarget organisms (NTOs). Globally, scientists, regulators, and stakeholders have expressed interest and concern for potential harm to NTOs, and the WHO recommends consideration of NTOs in GEM safety assessments [6]. Previous applications for the release of other GEM strains were critiqued for their lack of environmental impact consideration [11] and nontarget interactions with GEMs have been identified as a primary area for potential harm in other GEM release proposals and environmental risk assessments [12]. Although GEM technology requires careful consideration due to its novelty, current vector control methods are known to negatively impact NTOs, including use of bacterial larvicide [13], release of mosquitofish [14, 15], and spraying of insecticides [16–19]. A population replacement GEM release is intended to reduce environmental and NTO impacts. Therefore, examination of these interactions in a laboratory setting is prioritized by UCMI prior to the release of a GEM.

Predators of *An. coluzzii* are a group of NTOs expected to directly interact with GEMs released into their environment. Historically, consumption of genetically modified agricultural products has been an area of interest and concern to the public, and the perceived safety of this particular class of genetically modified organisms (GMOs) varies regionally [20–23]. Despite the extensive body of work that assesses the public perception and safety of GMO agriculture for human consumption, there is less available literature discussing the outcomes of GMO consumption by nonhuman organisms. No prior publication has examined potential effects arising from the consumption of genetically engineered *Anopheles* mosquitoes or any animal with a CAS-9 gene drive in vivo. The limited publicly available information regarding the impact of genetically engineered insects on their predators reports no negative impact from consumption of transgenic organisms. Published works include (1) a genetically engineered olive fly (*Bactrocera oleae*) and two of its predators: a *Pardosa* spider (Araneae, Lycosidae) and the rove beetle (*Aleochara bilineata*) [24], (2) genetically engineered *Aedes aegypti* and two predaceous *Toxorhynchites* species [25], and (3) genetically engineered *Ae. aegypti* and two of its predators: the guppy (*Poecilia reticulata*) and the American signal crayfish (*Pacifastacus leniusculus*) [26]. The final study was conducted by the biotechnology group Oxitec, though specific data from this study is not publicly available. In addition to these in vivo studies, bioinformatics and literature assessments of Cas9 endonuclease found negligible risk for human or animal allergenicity or toxicity [27]. No existing literature suggests that a GEM diet would harm or impact the growth of predators when compared with a wild-type (WT) mosquito diet.

The country of São Tomé and Príncipe (STP) has been identified as a potential release site for GEMs [28]. The country is made up of two islands located in the Gulf of Guinea off the coast of West Africa. *Anopheles coluzzii* is the only member of the *Anopheles gambiae* species complex present on the islands [29, 30].

São Tomé and Príncipe islands are geographically isolated and therefore exhibit unique and diverse ecology that is understudied. Species of birds, bats, crustaceans, amphibians, fish, and insects present on the islands are insectivorous or generalist predators that may predate upon *An. coluzzii*, but island-specific interspecies interactions are not well described [31]. Similar to all mosquitoes, *Anopheles* begin their lifecycle as aquatic larvae and pupae before emerging as terrestrial and aerial adults. Owing to environmental variability throughout the mosquito life cycle, predators of *Anopheles* are diverse and include both aquatic and terrestrial vertebrates and invertebrates [32].

The experiments described in this paper aim to investigate effects of a GEM diet rather than replicate natural *An. coluzzii* larval or adult predator–prey relationships. *Anopheles coluzzii* predator species in STP have not been described and even putative species are likely not easily managed in a laboratory setting. Therefore, two species not present in STP were selected as exemplar predators: an aquatic vertebrate, the mosquitofish *Gambusia affinis,* and a terrestrial invertebrate, the bold jumping spider *Phidippus audax*. These exemplar predator species represent predator groups present in STP, exhibit predictable growth patterns, and can be maintained in a laboratory setting.

The mosquitofish *G. affinis* has been introduced globally as a biological vector control agent owing to its proclivity for consuming mosquito larvae [33, 34]. *Gambusia affinis*’ success outside its native geographic range in the southeastern USA is attributed to its high climate tolerance, generalist diet, and intentional introduction for vector control [33, 35, 36]. *Gambusia affinis* has been introduced throughout much of mainland Africa, but has not been documented in STP [37]. The most closely related fish present in STP is *Aplocheilichthys spilauchen*, a fellow member of the Cyprinodontiformes order [31]. In addition, 25 members of the Gobidaee family are present on the islands, and several of these species are small generalist carnivores or insectivores that likely occupy a similar ecological niche to *G. affinis* [31]. *Gambusia affinis* was selected for this experiment due to its adaptable nature and predictable growth patterns in the laboratory.

The bold jumping spider *P. audax* (Family: Salticidae) is an active generalist predator that hunts using its excellent vision and ability to move or jump rapidly. *Phidippus audax* is not documented in STP, but 19 other members of the Salticid family are present on the islands [31]. *Phidippus audax’s* dynamic hunting strategy is representative of common predation techniques within the Salticid family. This hunting strategy allows Salticids, including *P. audax*, to consume a generalist insect diet, and makes these spiders well-equipped to feed on adult mosquitoes. *Evarcha culicivora,* a species of Salticid colloquially known as the vampire spider, is the only animal identified as a specialized *Anopheline* predator [32]. *Evarcha culicivora* is unique in its ability to identify and select blood fed female *Anopheles* as preferred prey [38, 39]. *Evarcha culicivora* is limited in range to the Lake Victoria region of East Africa, and its growth patterns have not been documented in depth [39]; therefore, this species was not considered as a candidate for our laboratory diet experiments. *Phidippus audax* was selected for the in vivo growth experiment owing to its broad distribution, availability in the USA, and extensive information on physiological development and life history [40].

Juveniles of each predator species were fed a diet of either GEM or WT *An. coluzzii* mosquitoes. Adult, semi-gravid female mosquitoes were selected as the experimental diet because the *Plasmodium*-refractory antibodies encoded within the gene construct are most strongly expressed in post-blood fed, semi-gravid female GEMs [8]. Larval, pupal, and newly emerged adult GEMs express two eye color reporter phenotypes: the fluorescent cyan blue color and a red color resulting from insertion of the transgene into the *cardinal* locus, a gene which produces pigment in the mosquito eye. These reporter phenotypes are not visible in adults over 48 h old. None of the phenotypic differences between GEM and WT *An. coluzzii* were expected to impact the growth of predators. Individual mortality, mass, and size were recorded weekly to compare growth between the two diet groups and to test the hypothesis that the GEM diet would not negatively impact predator growth.

## Methods

### Genetically engineered *Anopheles coluzzii*

The genetically engineered AcTP13 strain of *An. coluzzii* used in these experiments was developed and described by the James lab at UC Irvine [8]. The GEM strain was initially created via introgression of a drive system into the Mopti *An. coluzzii* strain (MRA-763) from BEI (BEI Resources, Manassas, VA, USA); therefore, the Mopti strain was used as the WT control for the studies described in this report. The AcTP13 GEM is equipped with two effector genes that produce single-chain variable fragment monoclonal antibodies that target *Plasmodium falciparum* at the ookinete and sporozoite stages [8]. These effector genes are most strongly expressed in adult females following a blood meal [8], and therefore adult semi-gravid or gravid *An. coluzzii* were used for predator diets.

### Predators

**Fig. 1:**
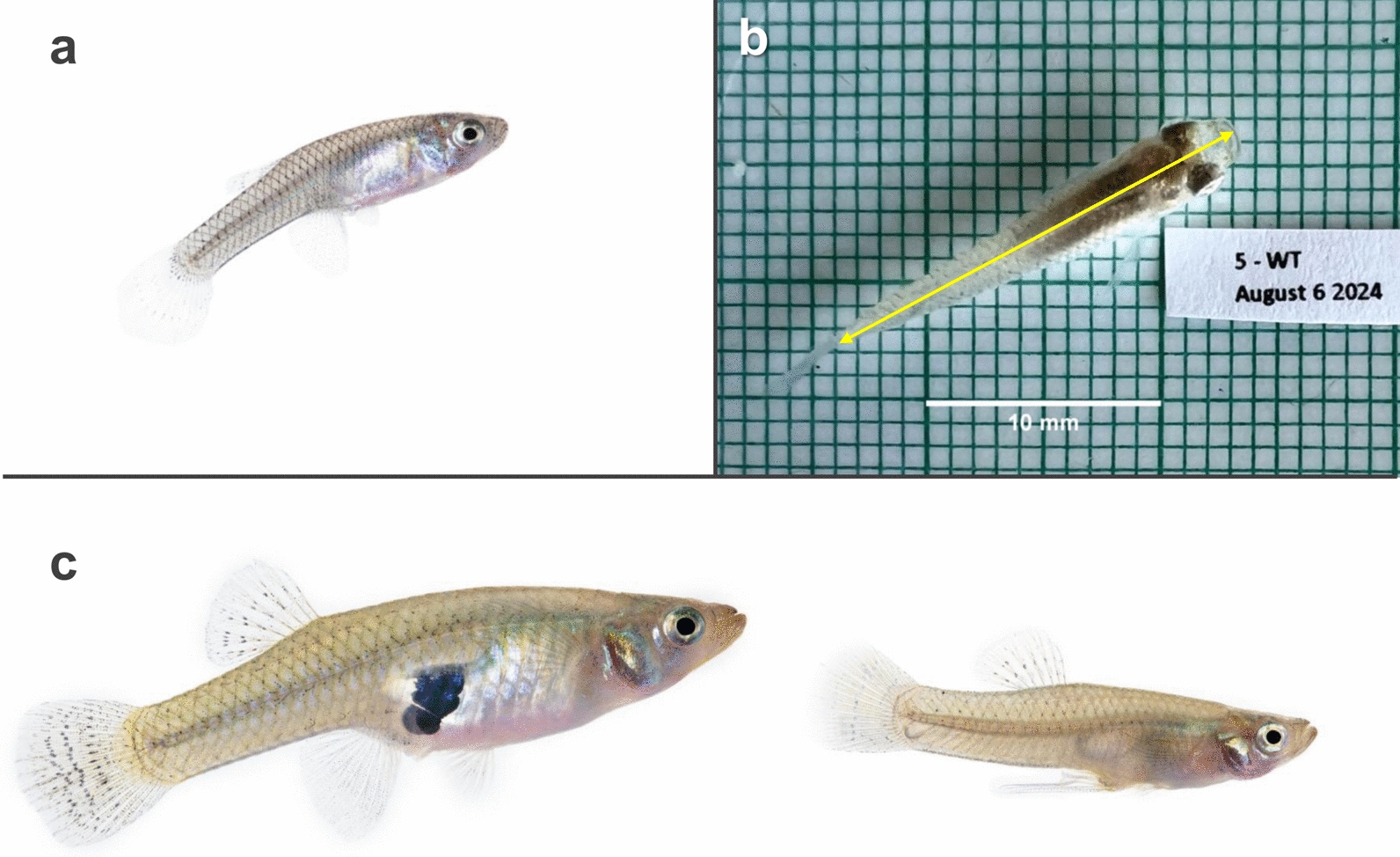
*Gambusia affinis* morphological characteristics and measurements. **a** Juvenile *G. affinis* of indistinguishable sex. **b** Fish length measured dorsally on a 1 mm × 1 mm grid from the tip of the mouth to the base of the caudal fin (yellow arrow). **c** Sexual differentiation in *G. affinis* shown in an adult female (left) displaying a black gravid spot and larger rounded body and adult male (right) displaying an elongated gonopodium (anal fin) and smaller narrow body (a and c not to scale)

**Fig. 2:**
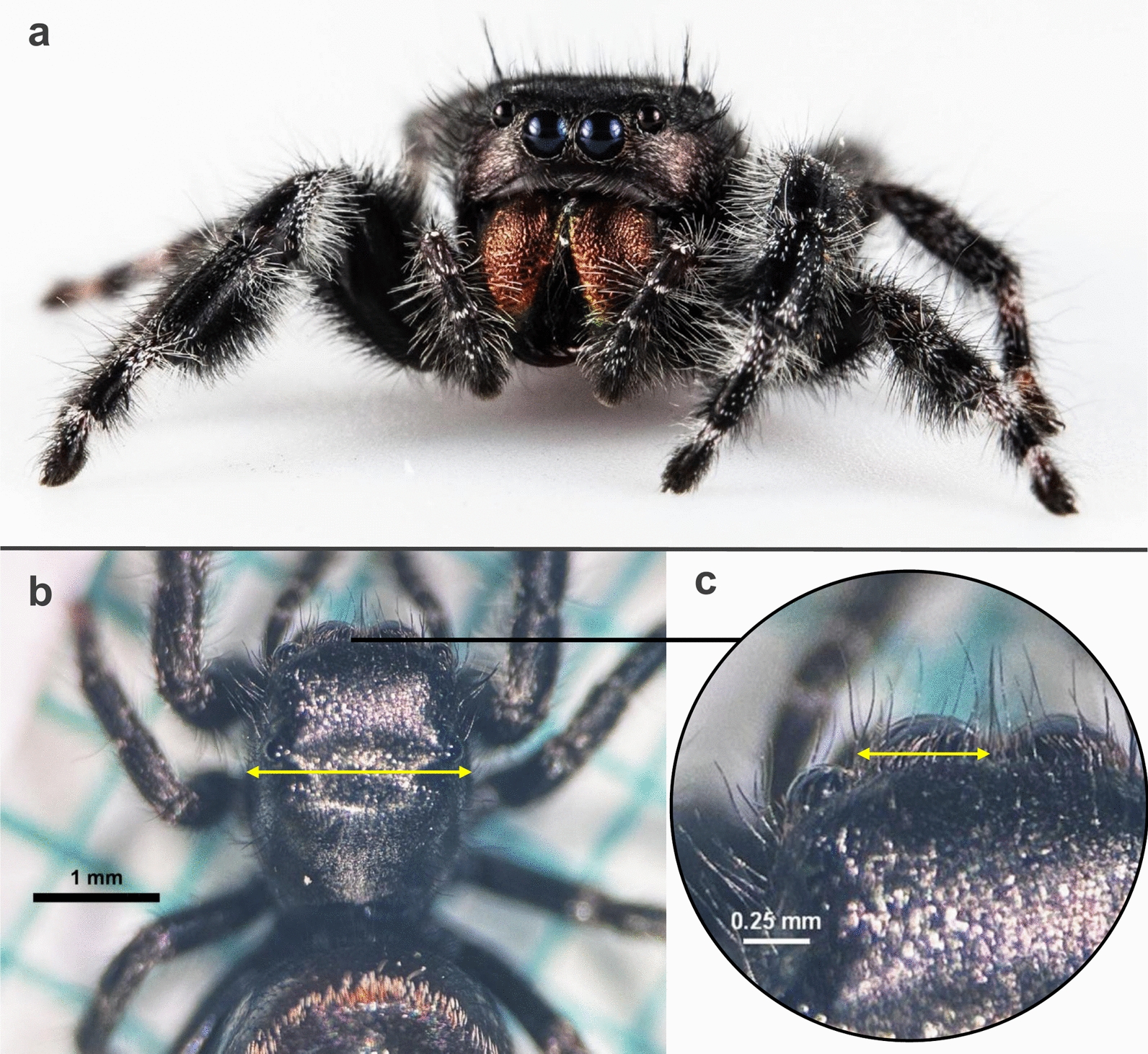
*Phidippus audax* characteristics and measurements. **a** Juvenile *P. audax* showing no phenotypic sex characteristics. Defined measurements of (**b**) carapace width and (**c**) anterior median eye diameter

Immature stages of two predator species, the mosquitofish *Gambusia affinis* (Fig. 1a) and the bold jumping spider *Phidippus audax* (Fig. 2a) were selected for this experiment. Immature stages were used because growth during this period occurs rapidly and predictably as individuals progress toward sexual maturity.

### Mosquitofish: *Gambusia affinis*

Gravid adult *G. affinis* fish were acquired from the Sacramento-Yolo Mosquito and Vector Control District (8631 Bond Rd Elk Grove, CA 95624). Each female was isolated in a 6-L birthing tank filled with conditioned tap water and equipped with an air stone for oxygenation. Each gravid female was placed in a screened-off birthing chamber within the tank. The screen prevented cannibalization by allowing fry to swim through while containing the adult female [41]. The adult female was removed from the chamber after giving birth. Fry were raised communally with their siblings for the first 2 weeks of life and fed Hikari First Bites Fish Food Granules (Petco, San Diego, California, USA) ad libitum daily. After 2 weeks, offspring from two separate mothers were divided evenly between two assigned diet groups, with offspring totaling 40 individuals per diet group. Fish were then reared individually in 700 ml plastic containers filled with 500 ml of conditioned tap water equipped with an air stone for oxygenation and a screened lid to prevent jumping.

Although adult *G. affinis* will readily feed on adult mosquitoes from the water’s surface, attempts to feed live adult mosquitoes to juvenile fish were unsuccessful owing to the juveniles’ small size. The *G. affinis* diet was designed to include high levels of expressed GEM antibodies rather than accurately reproduce predatory–prey dynamics between *G. affinis and An. coluzzii*. Therefore, adult mosquitoes were lyophilized and mixed with Hikari First Bites Fish Food Granules (Petco, San Diego, CA, USA) to prepare the *G. affinis* diet. Cages of adult mosquitoes containing a mixture of gravid, nongravid, and male mosquitoes were used to prepare the diet. *Anopheles* were blood-fed the evening before *G. affinis* diet preparation began. Cages of unsorted live adults were placed in a −20 °C freezer for 30 min. Each cage of frozen mosquitoes was funneled into a 15 ml tube stored at −20 °C for a maximum of 2 h. Each tube was lyophilized for 24 h using a Labconco FreeZone 2.5 Plus lyophilizer (Labconco, Kansas City, MO, USA). To ensure that the final GEM and WT diet mixtures contained the same percentage of gravid female mosquitoes, three parameters were recorded: (1) the average mass of individual lyophilized male, nongravid female, and gravid female mosquitoes for each strain, (2) the ratio of mosquito types (gravid females, nongravid females, and males) within each cage, and (3) the total mass of lyophilized mosquitoes per cage. To determine the average mass of each mosquito type, 50 individual males, nongravid, and gravid females from each strain were lyophilized separately, and the average mass per individual of each type was calculated. Prior to lyophilization, subsamples of approximately 150 mosquitoes from each cage were sorted into males, nongravid females, and gravid females to estimate the fractional ratio of mosquito type per cage. Finally, after lyophilizing each cage, all lyophilized mosquitoes were weighed to determine the total cage mass.

$${{\varvec{T}}}_{{\varvec{G}}{\varvec{F}}}=\frac{{f}_{GF}}{\left({\overline{m} }_{M}\cdot {f}_{M}\right)+\left({\overline{m} }_{GF}\cdot {f}_{GF}\right)+\left({\overline{m} }_{NG}\cdot {f}_{NG}\right)}\cdot C$$

The estimated total number of gravid females per cage ${({\varvec{T}}}_{{\varvec{G}}{\varvec{F}}})$ was determined using the average individual mosquito masses ($\overline{{\varvec{m}} }$) and fractional ratio (${\varvec{f}}$) of males (${\varvec{M}}$), nongravid females (${\varvec{N}}{\varvec{G}}$), and gravid females (${\varvec{G}}{\varvec{F}}$), and the total mass of the lyophilized cage (${\varvec{C}}$). Approximately 16 cages of each mosquito strain were processed to produce a mosquito mixture containing 68% gravid females and 32% males and nongravid females by mass. Although the diet mixtures also contained males and nongravid females, the number of gravid females was used as the basis for dietary composition because the gravid females express the genetically engineered proteins of interest to this study. The final diet mixture was prepared by grinding the lyophilized mosquitoes and mixing them with fish food to create a formulation of 10 gravid females per 14 mg of powdered food mixture. This mixture was 40% gravid female, 20% nongravid female and male, and 40% fish food by mass.

Once per week, mass and length measurements were collected for each fish. A week zero baseline measurement was taken before beginning the experimental diet. To measure mass, each fish was transferred from its enclosure to a 30 mm petri dish containing water on a pretared scale. A net was used to transfer the fish to prevent excess water from altering the fish mass. Fish mass was recorded to the nearest 0.1 mg. After weighing, each fish was photographed on a 1 mm × 1 mm grid background to measure its standard length. Standard length [42] was measured dorsally from the tip of the mouth to the base of the caudal fin using ImageJ software (Fig. 1b) [43, 44].

**Fig. 3:**
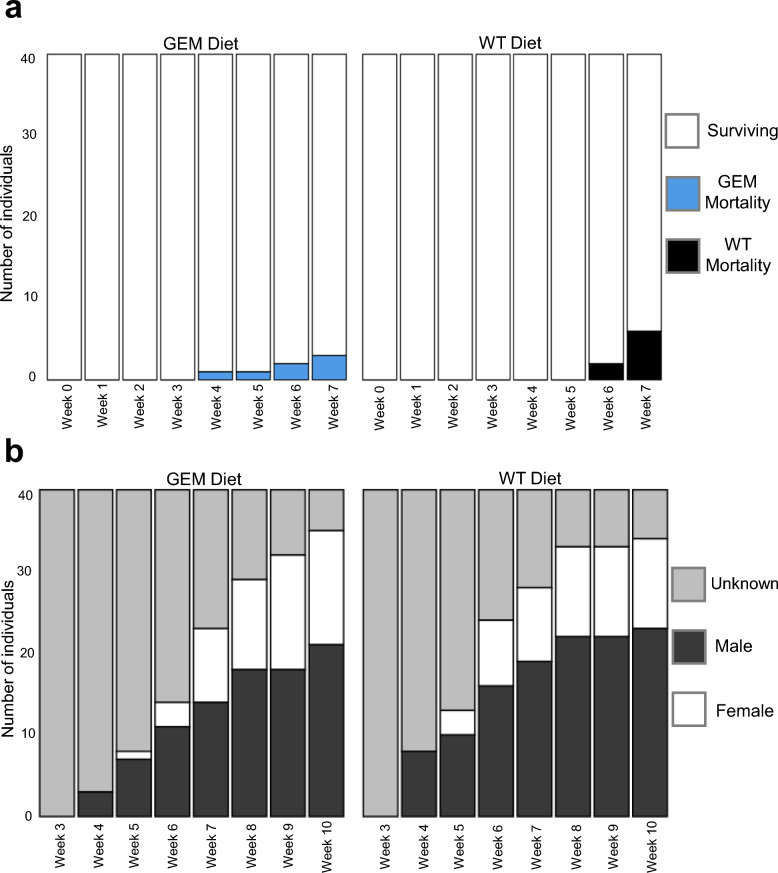
Weekly measurements of *G. affinis* development metrics. **a** Cumulative mortality rates for genetically modified mosquito (GEM) diet group (left) or wild-type (WT) diet group (right) and (**b**) sexual differentiation for GEM diet group (left) or WT diet group (right)

During the 7-week experiment, fish were fed a GEM or WT mosquito food mixture twice per week, with fish food provided on all other days. The daily ration of mosquito food mix and fish food was adjusted weekly to approximately 25% of the average fish body weight. On average, fish consumed 14.5 gravid females per week. Water in each container was replaced prior to the twice-weekly mosquito feedings. Fish were monitored daily for mortality (Fig. 3a) and observed twice weekly for signs of sexual differentiation (Fig. 3b). Fish displaying a gravid spot were classified as female, while those with an elongated gonopodium were classified as male (Fig. 1c). After the initial 7-week period, fish were reared for an additional 3 weeks and fed Hikari fish bites to allow more individuals to achieve sexual differentiation (Fig. 3b).

### Jumping spiders:* Phidippus audax*

Two gravid *P. audax* females were purchased from jumpingspidersforsale.com (Garden Grove, CA, USA). Each female was housed in a 101.6 mm × 101.6 mm × 128.6 mm enclosure and fed a diet of crickets and fruit flies twice per week. Hatchlings were kept in the same enclosure as the mother until they began to leave the nest, approximately 1 month after hatching. The 59 total hatchlings from two mothers were placed in individual enclosures and distributed evenly between the two diet groups. The sex ratio was initially unknown as juvenile male and female jumping spiders are morphologically indistinguishable (Fig. 2a).

Individual enclosures measured 12.5 × 5.5 × 5.5 mm and contained a Plaster of Paris substrate and cork bark affixed to one side. Small, screened ventilation holes were provided on the sides and lids of the container, with a larger plugged hole at the base of the enclosure to allow for the introduction of mosquito prey. Enclosures were sprayed with water three times per week. Prior to the start of the experiment, each juvenile spider was fed five fruit flies per week. A week 0 baseline measurement was taken before the initial introduction of GEM or WT mosquito diets.

During the experiment, spiders were exclusively fed ten live, half-gravid, female GEM or WT *An. coluzzii* mosquitoes once per week for 11 weeks. A total of 30 spiders were used for each diet group. Spiders and remaining mosquitoes were lightly anesthetized with CO_2_ and removed from their enclosures 48 h after feeding. The number of uneaten mosquitoes remaining in the enclosure was recorded and all mosquitoes, mosquito carcasses, and spider molts were removed. The anesthetized spiders were weighed on a tared scale to the nearest 0.1 mg and photographed on a 1 mm × 1 mm grid background positioned under a microscope for carapace width and eye diameter measurements. Photos were analyzed using ImageJ software to acquire the carapace width (Fig. 2b) and anterior median eye diameter (Fig. 2c) [40, 43]. Mortality was monitored twice per week.

After the completion of the feeding experiment, *P. audax* were housed individually for an additional 3 weeks and fed a diet of 15 fruit flies per week. Spiders were then euthanized and preserved in ethanol. Preserved *P. audax* individuals were examined morphologically to determine sex. Males were identified via the presence of palpal bulbs on the pedipalps, and females were identified via the presence of an epigynum on the underside of the abdomen. Individuals that did not reach penultimate or adult instars had indistinguishable morphological sex characteristics at the time of euthanasia and were recorded as unknown sex.

### Analyses

Standard deviation (SD) was calculated to assess differences in the GEM and WT groups. A two-way analysis of variance (ANOVA) was used to test for effects of diet group, sex, and their interaction on predator growth [45]. Following ANOVA results, Tukey’s honest significant difference post-hoc test was used to determine if growth differed significantly between sexes (female, male, or unknown) and to determine if growth differed between individuals of the same sex across the two diet groups. Fisher’s exact test [46] was used to determine if mortality rates differed significantly, and a Chi-squared test [47] was used to determine if sex ratios differed significantly. All analyses were conducted in R version 4.4.2 [48] using the rstatix, dplyr, broom, and purr packages for statistical generation and analysis [49–52], ggplot2 package for plot creation [53], and ggpubr for statistical integration in plots [54]. A *P*-value of < 0.05 was considered statistically significant.

## Results

### Gambusia affinis

The 40 juvenile fish in each diet group followed expected growth patterns as they matured throughout the 7-week experiment. Mortality was low and did not differ significantly for the two *G. affinis* diet groups (*P*-value = 0.481) (Fig. 3a). A total of six fish died in the WT diet group and three fish died in the GEM diet group during the experiment.

Fish sex ratios did not differ significantly between diet groups (*P*-value = 0.933). Fish began sexually differentiating in week 4 of the experiment, after which approximately 13 individuals differentiated per week. Fish that did not sexually differentiate before the experiment concluded remained in their individual enclosures until sex could be determined or until they died. The sex of individuals that did not reach maturity was recorded as unknown. The final GEM group sex distribution was 60% male, 27.5% female, and 12.5% unknown. The final WT group sex distribution was 60% male, 25% female, and 15% unknown (Fig. 3b).

**Fig. 4:**
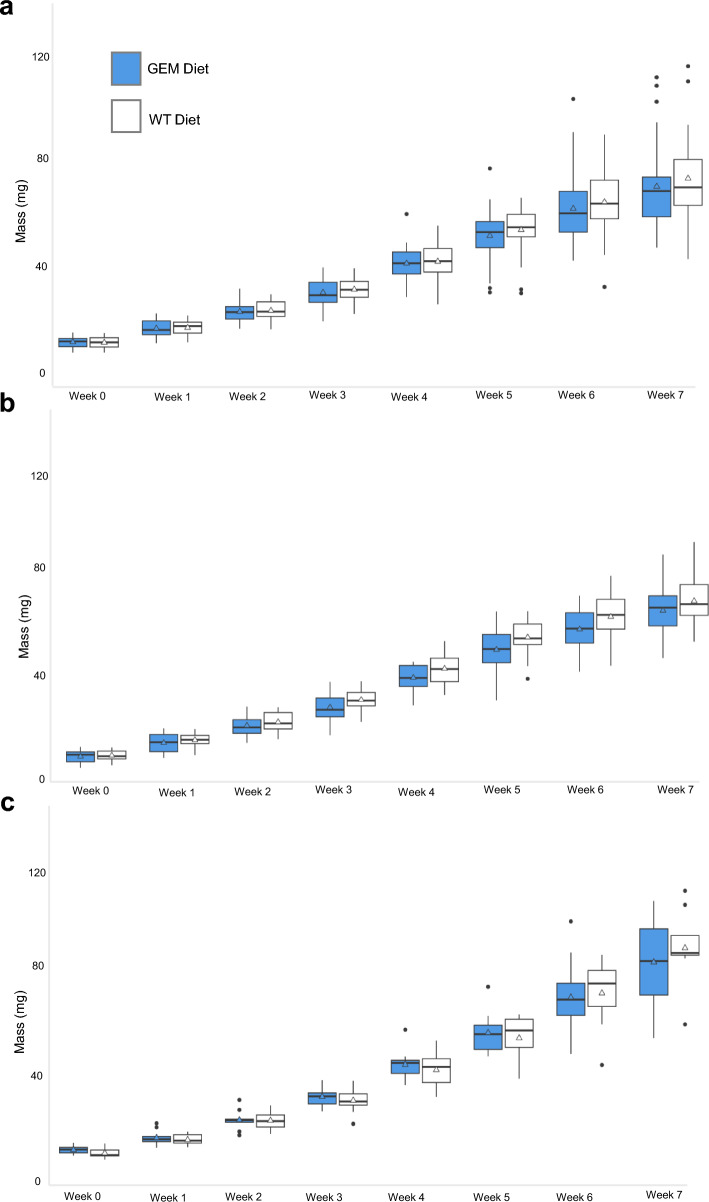
Mean mass for *G. affinis* diet groups. Boxplot of mass across **a** all individuals including males, females, and unknown individuals, **b** males, and **c** females. Outliers are represented as individual black points. Median values are indicated with a black crossbar, and mean values are indicated with a triangle. An adjusted *P*-value of < 0.05 was considered statistically significant

No significant difference in *G. affinis* mass was observed between diet groups for any week of the experiment (Supplementary Table S1). In addition to overall diet group comparison, the mass of females, males, and unknown individuals across diet groups showed no significant differences, except for male mass in weeks 4 and 5 (adjusted *P*-values = 0.0319, 0.0336) (Supplementary Table S2). *Gambusia affinis* mass did differ significantly among sexes across diet groups, with significant differences between sexes in weeks 4–7 of the experiment. These differences were significant between males and females and females and unknown individuals (Supplementary Table S3). Overall fish mass increased by an average of 5.85 times for the GEM diet group and 6.08 times for the WT diet group over the 7 weeks (Fig. 4a). In males, this increase was 5.22 times in the GEM group and 5.33 for the WT group (Fig. 4b). In females, this increase was 7.23 times in the GEM group and 8.25 times for WT group (Fig. 4c). Mass became more variable within each diet group over time. This increase in variability was consistent across both diet groups (Fig. 4a); week 0 standard deviation for males, females, and unknown individuals combined was 1.85 mg for the GEM diet group and 2.02 mg for the WT diet group and by week 7, the standard deviation was 17.79 mg for the GEM diet group and 15.32 mg for the WT diet group. When the experiment began, the variability was similar across sexes in both diet groups, but female mass became more variable than male mass over time. Week 0 standard deviation was 2.06 for GEM and 1.60 for WT males (Fig. 4b), and 1.53 for GEM and 1.99 for WT females (Fig. 4c), whereas, week 7 standard deviation was 8.38 for GEM males and 8.42 for WT males (Fig. 4b), and 20.28 for GEM and 15.64 for WT females (Fig. 4c).

**Fig. 5:**
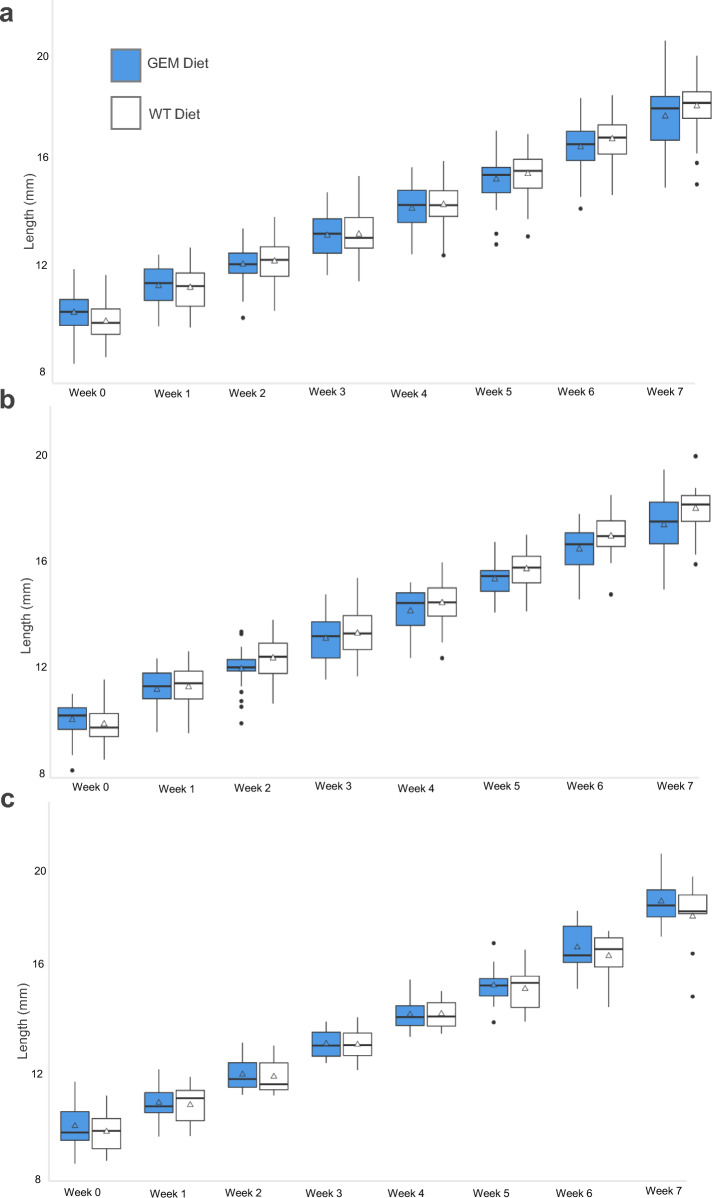
Mean length for *G. affinis*. Boxplot of length across (**a**) all individuals including males, females, and unknown individuals, (**b)** males, and (**c**) females. An adjusted *P*-value of < 0.05 was considered statistically significant. Median values are indicated with a black crossbar, and mean values are indicated with a triangle. Outliers are represented as individual black points

Similarly, the average fish length was not significantly different between GEM and WT diet groups for any week (Supplementary Table S1 and Table S2). Fish length differed most between diet groups in week 0, prior to the first administration of the variable experimental diet (*P*-value = 0.0554). The fish steadily increased in length over time, and by week 7, the length of the entire GEM diet group increased by 1.85 times and the length of the entire WT diet group increased by 1.75 times (Fig. 5a). The increase in length was similar across sexes in both diet groups, and only differed significantly between males and females in week 7 (*P*-value = 0.017823996) (Supplementary Table S3). Male length increased an average of 1.74 times in the GEM group and 1.83 times in the WT group (Fig. 5b). Female length increased an average of 1.87 times for the GEM group and 1.85 times for the WT group (Fig. 5c). Variance in fish length was consistent throughout the experiment; standard deviation ranged from 0.724 to 1.295 within the entire GEM diet group, and from 0.772 to 1.13 within the entire WT diet group (Fig. 5a). Male variance was between 0.68 and 1.13 in the GEM group and 0.73 and 0.98 in the WT group (Fig. 5b). Female variance ranged between 0.58 and 0.94 in the GEM group and 0.58 and 1.45 in the WT group (Fig. 5c).

### Phidippus audax

**Fig. 6:**
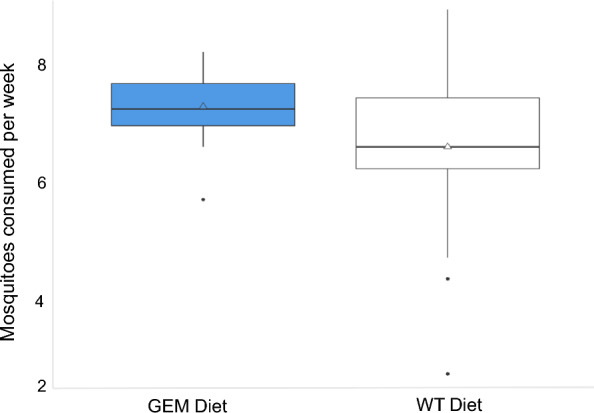
Mean weekly mosquito consumption for *P. audax* over the course of 11 weeks. Median values are indicated with a black crossbar, and mean values are indicated with a triangle. Outliers are represented as individual black points

**Fig. 7:**
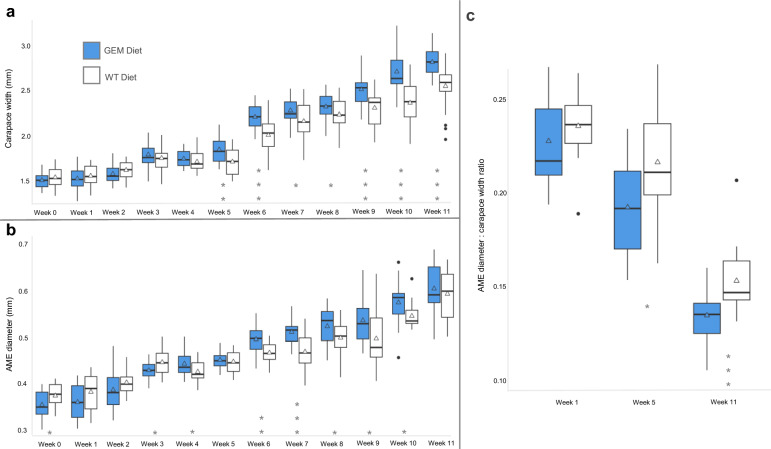
Weekly measurements of *P. audax* size metrics. Boxplots of **a** mean carapace size for genetically engineered mosquitoes (GEM) and wild-type (WT) diet groups **b** mean anterior median eye (AME) diameter, **c** mean AME-to-carapace-width ratio. Median values are indicated with a black crossbar, and mean values are indicated with a triangle. Outliers are represented as individual black points. * Adjusted *P*-value < 0.05, **Adjusted *P*-value < 0.01, *** Adjusted *P*-value < 0.001

The *P. audax* diet experiment began with 30 juvenile spiders in the GEM group and 29 in the WT group. Mortality was low for *P. audax*, and did not differ significantly between the dietary groups, with two deaths in the WT group and zero in the GEM group (*P*-value = 0.237). *Phidippus audax* fed a GEM diet consumed an average of 7.3 mosquitoes per week, 10.61% more than the WT diet group average of 6.6 mosquitoes per week (*P*-value = 0.0162) (Fig. 6). Carapace width did not differ significantly for weeks 0–4 of the experiment (*P*-values ≥ 0.05), but did significantly differ weeks 5–11 (Fig. 7a and Supplementary Table S4). This difference was significant beginning in week 5 for females but was not significant until week 10 for males (Supplementary Table S5). The average anterior median eye (AME) size differed significantly between diet groups in weeks 0, 3, 4, and 6–10, but not in weeks 1, 2, 5, or 11 (Supplementary Table S4). For both groups, AME size increased over time (Fig. 7b). The ratio of AME size to carapace width decreased over time for both species, with the GEM AME-to-carapace-width ratio significantly smaller in weeks 3, 5, 7, 10, and 11 (Fig. 7c). Some individuals were excluded from certain weekly AME and carapace measurements if body positioning in the photograph did not allow for visualization of AME or carapace borders, resulting in variable weekly degrees of freedom (Supplementary Table S4).

**Fig. 8:**
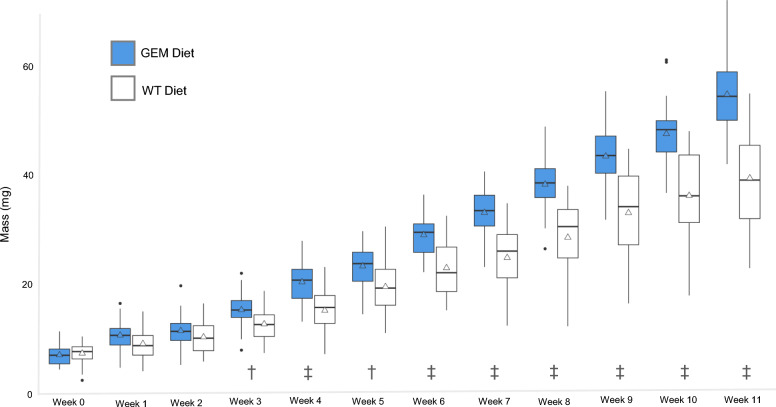
Weekly mean mass of *P. audax*. Median values are indicated with a black crossbar, and mean values are indicated with a triangle. Outliers are represented as individual black points. † Adjusted *P*-value < 0.01, ‡ Adjusted *P*-value < 0.0001

The mean spider mass for each group did not differ significantly in weeks 0–3 of the experiment. The GEM diet group was significantly larger (adjusted *P*-value < 0.05) than their WT diet counterparts in weeks 3–11, with the difference in mass generally increasing in significance over time (Fig. 8 and Supplementary Table S4). This significant difference was true for both males and females compared across the diet groups (Supplementary Table S5). *Phidippus audax* in the GEM group weighed more and grew more on average per week compared with those fed WT mosquitoes, and by week 11, the GEM diet group was 1.37 times larger than the WT group. Spider mass variance also increased over time for both diet groups at similar rates (Fig. 8). Week 0 mean spider mass was 8.45 mg ± standard deviation of 1.87 mg for the GEM group and 8.74 mg ± 1.99 mg for the WT group, and week 11 mean mass was 54.14 mg ± 7.00 mg for the GEM group and 39.45 mg ± 8.49 mg for the WT group.

**Fig. 9:**
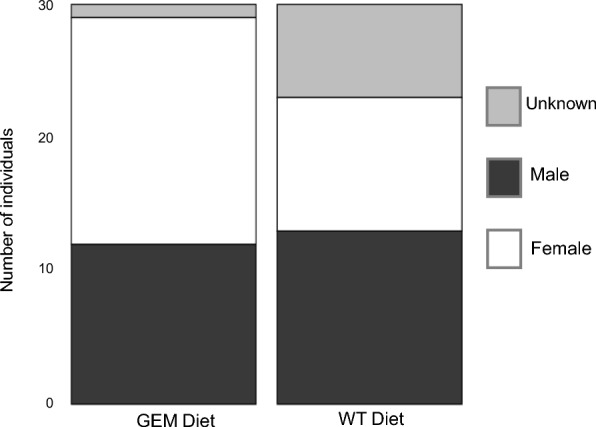
Sex ratio of *P. audax* fed a GEM diet (left) or WT diet (right)

No parameter for spider growth (spider mass, carapace width, AME diameter, and AME-to-carapace-width ratio) differed significantly between males and females for any week of the experiment according to Tukey’s test (adjusted *P*-values ≥ 0.05) (Supplementary Table S6). Male and female spiders had significantly higher mass than spiders of unknown sex in weeks 8, 9, 10, and 11 and significantly larger carapace width in weeks 8, 9, and 11 (Supplementary Table S6). The sex ratio of *P. audax* did not vary significantly between the two groups (*P*-value = 0.06684), with GEM sex ratio of 40% male, 56% female, and 3% undetermined, and WT sex ratio of 45% male, 34% female, and 21% undetermined (Fig. 9). Although the sex ratios were not significantly different, there were seven times as many individuals with indeterminable sex in the WT group.

## Discussion

The effects of a GEM diet varied between the two predator groups but showed no negative impact on any measured parameters including mass, size, and mortality. No significant differences were found in fish length, mass, or mortality between experimental and control groups of *Gambusia affinis*. However, by the end of the experiment, *Phidippus audax* fed a GEM diet had larger average carapace width and mass, a higher proportion of sexually mature individuals, and an eye-to-carapace size ratio indicating greater developmental progression compared with those fed WT mosquitoes.

Differences in experimental design may explain the contrasting results between the two species. *Gambusia affinis* were fed lyophilized mosquitoes supplemented with fish food, excluding behavioral or phenotypic influences of the mosquito strains on growth. In contrast, *P. audax* were fed live adult mosquitoes, suggesting that the observed growth differences could be attributed to variations in mosquito phenotype or behavior.

Importantly, no increase in mortality or decrease in average size and mass was observed in either predator species fed a GEM diet. These findings align with previous studies showing that CAS-9 gene-drive systems and fluorescent proteins pose no significant allergenic or toxicity risks when consumed [27, 55]. These results also indicate that the two effector gene antibody products, expressed in semi-gravid female GEMs, are not toxic.

*Gambusia affinis* growth did not differ significantly between diet groups, but the mass of fish within each diet group became increasingly variable over time (Fig. 4). This is likely due to divergence in growth patterns between subadult males and females. Juvenile male and female *G. affinis* are monomorphic (Fig. 1a) but become sexually dimorphic as they mature, with females growing larger than males (Fig. 1c). The pace of growth slows for both sexes after maturity, and growth patterns diverge, with male *G. affinis* growth stopping entirely (Fig. 4b) while females exhibit indeterminate growth (Fig. 4c) [33]. *Gambusia affinis* siblings of unknown sex were randomly distributed between the diet groups before the experiment began, and therefore, the sex ratio did not differ significantly between GEM and WT diet groups (Fig. 3b). When preparing the *G. affinis* diet, the average mass of lyophilized *An. coluzzii* males, nongravid, and gravid females did not vary between GEM and WT mosquito strains.

*Phidippus audax* fed a GEM diet consumed an average of 10% more mosquitoes per week (Fig. 6). This increase in mosquito consumption likely contributed to larger carapace size (Fig. 7), higher mass (Fig. 8), and increased maturity rates in GEM-fed *P. audax* (Fig. 9)*.* When assessed using an ANOVA model, sex did not appear to significantly influence growth (Supplementary Table S6), but diet impacted the growth of all sexes over time (Supplementary Table S5). The increased vulnerability of GEMs to predation may stem from impaired predator avoidance abilities or phenotypic traits that make them more attractive to predators. This finding is particularly important as it suggests that GEMs may exhibit differences in predator avoidance within natural environments. It highlights an aspect of GEM fitness that conventional laboratory assessments may not adequately capture.

Because molts were not tracked for individual spiders, the lower average mass observed in the WT diet group could be attributed to slower maturation or smaller size at the same developmental instars. Multiple growth trends observed between *P. audax* diet groups suggest that, in addition to growing larger, more individuals fed a GEM diet reached maturity upon completion of the study. Unlike juvenile *G. affinis*, immature female and male *P. audax* of the same instar were expected to be of similar size [40]. Results of the two-way ANOVA confirmed that mass did not differ significantly between male and female spiders. *Phidippus audax* of undetermined sex were significantly smaller than male and female *P. audax* across diet groups in later weeks of the experiment. The sex of *P. audax* can only be determined during the penultimate and adult instars, which require at least four molts. Individuals with undetermined sex died before reaching this stage. A higher proportion of *P. audax* fed a WT diet had undetermined sex at the conclusion of the experiment. Juvenile *P. audax* also exhibit proportionally larger eyes relative to their body size compared with adults, making the AME diameter-to-carapace width ratio a key indicator of development. As spiders progress to later instars, this ratio decreases [40]. Spiders fed a GEM diet showed a significantly smaller average AME-to-carapace ratio in weeks 10 and 11, further supporting the observation that these individuals reached later instars more quickly than their WT-fed counterparts. The WT-fed *P. audax’s* higher AME-to-carapace ratio, lower proportion of sexual maturity, and smaller average body size indicate these spiders grew less and potentially matured more slowly than their counterparts that were fed a GEM diet.

Within these experiments, *An. coluzzii* made up overly representative proportions of predator diets. Both *G. affinis* and *P. audax* are generalist predators with varied diets in nature. Although *G. affinis* can consume large quantities of mosquitoes, they cannot survive if mosquitoes are their only food source [33]. In preliminary work with *P. audax*, unfed adult mosquitoes appeared to lack adequate nutritional content to sustain spider growth, but postblood fed, semi-gravid female mosquitoes were able to sustain *P. audax* growth, likely owing to their increased nutritional content [56].

In nature, adult *Anopheles* mosquitoes generally do not make up a large percentage of a generalist predator‘s diet due to their low energy profitability—for many predators, the gain in nutrition from *Anopheles* consumption is too low to support the energy expenditure required to hunt them [32]. In anecdotal insectary and experimental observations, we noted that adult GEMs were less active and easier to collect using aspirators compared with WT individuals. Salticid spiders hunt by jumping at high speeds over short distances, a strategy that requires high energy input relative to spider body size [57]. When a jumping spider misses a target, the lack of nutritional gain is compounded by the energy expended through high-speed jumping. If the successful capture of GEM mosquitoes required less energy expenditure for *P. audax*, it may explain why spiders fed GEMs grew larger over the course of the experiment. If predators require less energy expenditure to hunt GEM mosquitoes, GEM fitness may be directly impacted postrelease by higher rates of predation.

Physical differences between GEMs and WT mosquitoes may contribute to GEM vulnerability to predation, but the precise difference that accounts for this increased vulnerability is, at present, unknown. UCMI’s GEMs differ from WT *An. coluzzii* through three key modifications: a cyan fluorescent marker protein, the CAS-9 gene drive, and two effector genes. These effector genes encode antibodies targeting *Plasmodium falciparum* at different stages of the parasite’s development in adult female mosquitoes [8]. The cyan fluorescent marker protein (CFP) and red eye phenotype displayed in GEM mosquitoes may contribute to increased predation. GEM larvae, pupae, and adults less than 48 h old display a red-eye phenotype that fluoresces under ultraviolet (UV) light because of disruption of the cardinal gene and insertion of CFP [8]. As visual hunters, Salticid spiders detect a wide range of light spectra, including UV wavelengths outside the range of human perception [58], and the Salticid *E. culicivora* is able to distinguish phenotypic differences between mosquitoes such as sex and blood fed status [38]. Therefore, *P. audax* may be able to perceive the CFP signal in adult GEMs over 48 h postemergence, when the CFP signal and red eye phenotype are no longer visible to the human eye. It is also possible that the red eye phenotype or CFP expression affects the visual acuity of mosquitoes. In *Drosophila*, the *cd1* cardinal gene mutation that causes a red eye phenotype can also cause negative neurological impacts [59]. If effects are similar in GEM *An. coluzzii*, neurological issues could impact the GEMs’ ability to visually respond to attempted predation.

## Conclusions

The objective of this study was to assess if GEM consumption negatively impacted predators. We found no evidence that survival or growth in the spider *P. audax* or the fish *G. affinis* was negatively impacted by ingestion of the transgenic AcTP13 strain of *An. coluzzii*. Our results do suggest that differences in GEM behavior may decrease their ability to avoid spider predation. Further analysis of GEM predator avoidance may be an important avenue for future study.

## Supplementary information

Additional file 1.

Additional file 2.

Additional file 3.

Additional file 4.

Additional file 5.

Additional file 6.

## Data Availability

Data supporting the main conclusions of this study are included in the manuscript.
